# Pse-Analysis: a python package for DNA/RNA and protein/peptide sequence analysis based on pseudo components and kernel methods

**DOI:** 10.18632/oncotarget.14524

**Published:** 2017-01-05

**Authors:** Bin Liu, Hao Wu, Deyuan Zhang, Xiaolong Wang, Kuo-Chen Chou

**Affiliations:** ^1^ School of Computer Science and Technology, Harbin Institute of Technology Shenzhen Graduate School, Shenzhen, Guangdong, China; ^2^ Key Laboratory of Network Oriented Intelligent Computation, Harbin Institute of Technology Shenzhen Graduate School, Shenzhen, Guangdong, China; ^3^ Gordon Life Science Institute, Boston, Massachusetts, USA; ^4^ School of Computer, Shenyang Aerospace University, Shenyang, Liaoning, China; ^5^ Key Laboratory for Neuro-Information of Ministry of Education, School of Life Science and Technology, Center for Informational Biology, University of Electronic Science and Technology of China, Chengdu, China

**Keywords:** sequence analysis, pseudo components, support vector machine, genome/proteome analysis

## Abstract

To expedite the pace in conducting genome/proteome analysis, we have developed a Python package called Pse-Analysis. The powerful package can automatically complete the following five procedures: (1) sample feature extraction, (2) optimal parameter selection, (3) model training, (4) cross validation, and (5) evaluating prediction quality. All the work a user needs to do is to input a benchmark dataset along with the query biological sequences concerned. Based on the benchmark dataset, Pse-Analysis will automatically construct an ideal predictor, followed by yielding the predicted results for the submitted query samples. All the aforementioned tedious jobs can be automatically done by the computer. Moreover, the multiprocessing technique was adopted to enhance computational speed by about 6 folds. The Pse-Analysis Python package is freely accessible to the public at http://bioinformatics.hitsz.edu.cn/Pse-Analysis/, and can be directly run on Windows, Linux, and Unix.

## INTRODUCTION

With the explosive growth of biological sequences in the post-genomic age, we are facing a lot of binary classification problems. For DNA/RNA sequences, these problems are about how to identify the recombination spots [1–4], nucleosome positioning [5–9], promoters [10], microRNA precursors [11–13], enhancers [14, 15], translation initiation sites [16, 17], various PTRM (postpost-replication modification) sites in DNA [18] and PTCM (post-transcriptiom modification) sites in RNA [19, 20], RNA pseudouridine sites [21], DNA origin of replication [22, 23], adenosine to inosine editing sites in RNA [24], and many more other topics as mentioned in a recent review article [25].

For protein/peptide sequences, they are about how to identify various PTM (Posttranslational Modification) sites [26–42], anticancer peptides [43, 44], interactions between drugs and target proteins [45–49], PPI (protein-protein interaction) [50]. PPBS (proire-protein binding sites [51, 52], as well as a long list of references cited in a recent comprehensive review [53].

It is quite laborious even if using computational approches to deal with these problems since the development of each computational predictor needs to undergo the following five steps [54]: (1) benchmark dataset preparation, (2) optimise sample formulation, (3) optimize operation engine, (4) conduct cross-validations, and (5) establish a web-server. Each of the five procedures is time-consuming and tedious, particularly in how to select the optimal parameters [55–60] for the samples concerned and for the operation engine adopted.

To speed up such processes, we are to propose a Python package called Pse-Analysis, which is based on the framework of LIBSVM [61] and which can automatically generate the predictor desired by users. The users only need to input their benchmark dataset and the query biological sequences, followed by getting their desired results from the output of the Pse-Analysis system. All the tedious things in the aforementioned steps (2)–(5) can be totally skipped and leave them to be fulfilled by the computer.

## RESULTS AND DISCUSSION

**Figure 1:**
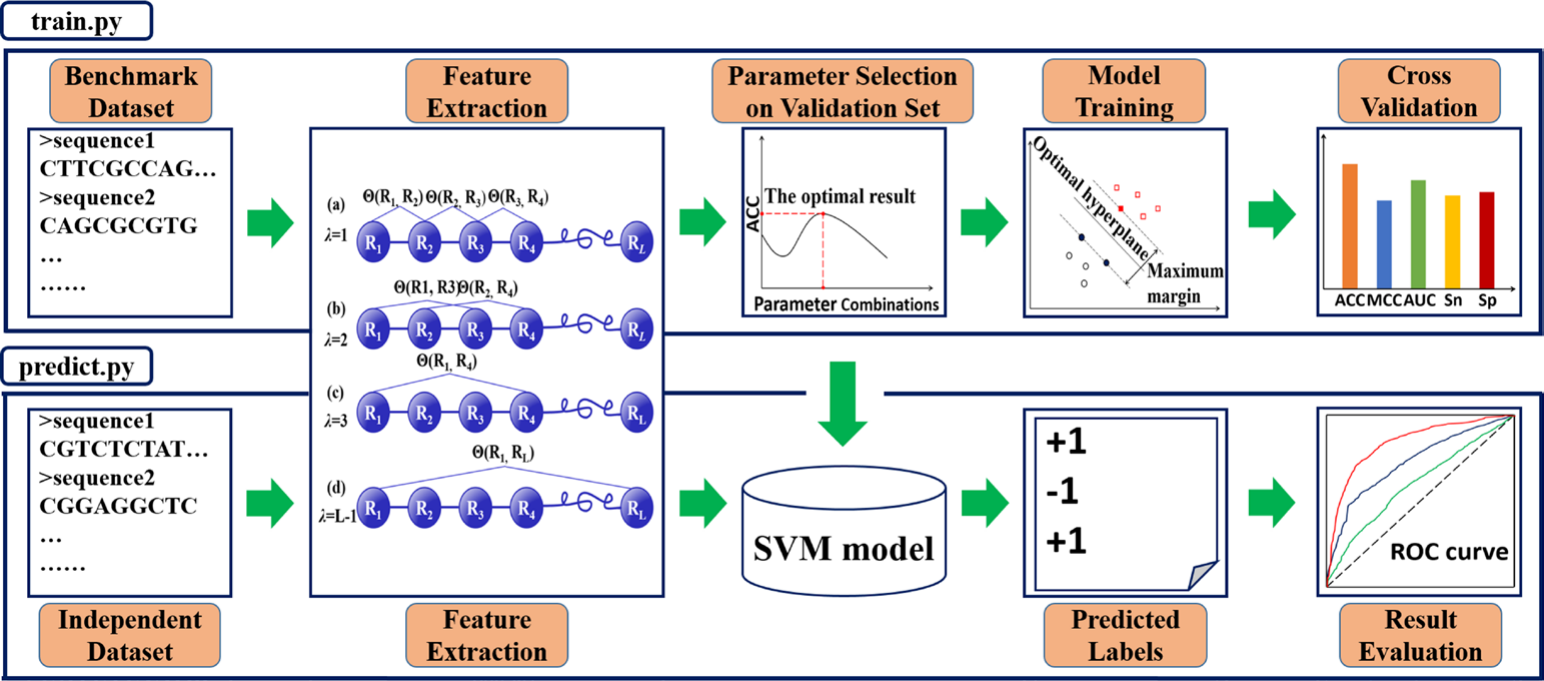
The flowchart of Pse-Analysis Python package The “train.py” script is for training the predictive model based on the benchmark dataset submitted by the user. It contains four procedures; i.e., feature extraction, parameter selection, model training, and cross validation. The “predict.py” is for using the trained model to predict the query samples and evaluate their prediction quality by a set of widely used metrics Acc, MCC, Sn, Sp [25], and AUC [68].

A powerful Python package, called Pse-Analysis, has been developed, and its web-server established at http://bioinformatics.hitsz.edu.cn/Pse-Analysis/. It is formed by two important parts: one is “train.py”, and the other is “predict.py” (Figure 1).

The “train.py” is designed for training a Support Vector Machine (SVM) model. It includes four steps: (1) feature extraction, (2) parameter selection, (3) model training, and (4) cross validation.

The “predict.py” is to generate the output. Note: the meaning of the “output” here is not limited in the predicted results for the original query biological sequence data submitted along with the benchmark dataset, but also include an optimal predictor. Users can directly apply it on various relevant problems, substantially saving a lot of time to repeat tedious for developing an effective predictor.

For instance, it is a very important task to effectively predict nucleosome positioning in genomes. To deal with the problem, Guo et al. [7] had praiseworthily developed a predictor called iNuc-PseKNC by going thru all the five procedures described in the Introduction section. Now, with the Pse-Analysis package, what we need to do is just to input the benchmark dataset used by Guo et al. [7] into the package, and Pse-Analysis will automatically do all the remaining jobs: optimising sample formulation; optimising operation engine; conducting cross-validations; and forming a web-server that is fully equivalent to the iNuc-PseKNC of [7].

**Figure 2:**
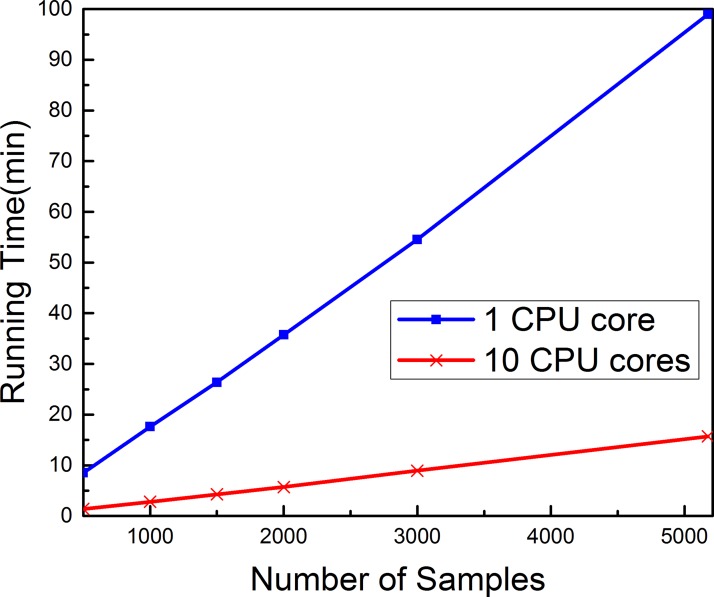
The computational cost of Pse-Analysis can be significantly reduced by using multiprocessing technique The blue curve reflects the computational time time for the parameter optimization process when using Pse-Analysis of one CPU core to process the five subsets for nucleosome positioning prediction of *Caenorhabditis elegans* [7], while the red curve reflects the corresponding computational time when using Pse-Analysis of ten CPU cores to do the same.

The computational speed in optimizing many different parameters is a bottleneck for the efficiency of the Pse-Analysis platform. In this regard, the multiprocessing technique has been applied to significantly speed up the computational processes. It has been shown when dealing with the above case that the computing time for the parameter optimization process can be reduced by 6 folds when using 10 cores instead of a single core, as shown in Figure 2.

As pointed out in a comprehensive review paper [25], the general form of PseKNC (pseudo K-tuple nucleotide composition) can cover all the existing feature vectors for DNA/RNA sequences. And the general form of PseACC (pseudo amino acid composition) can cover all the existing feature vectors for protein/peptide sequences [54, 56]. Particularly, the very powerful web-server Pse-in-One [60] developed recently not only can cover all the existing feature vectors for DNA/RNA and protein/peptide sequences, but also can cover those defined by the users themselves. Accordingly, the pseudo components in the Pse-Analysis package have virtually covered all the feature vectors for DNA/RNA or protein/peptide sequences.

## MATERIALS AND METHODS

### Feature extraction

In the Pse-Analysis, various state-of-the-art algorithms are employed, including pseudo k-tuple nucleotide composition (PseKNC) [25, 57–59, 62] and pseudo amino acid composition (PseAAC) [56, 60, 63–67] for extracting the features of DNA, RNA, and protein sequences, respectively. The details of these algorithms have been clearly elaborated in the aforementioned papers, and hence there is no need to repeat here.

### Parameter selection

The aforementioned algorithms contain some uncertain parameters, such as *k*, λ, and *w*. All these parameters are automatically determined by train.py in processing the benchmark dataset submitted by users. The concrete process is to optimize the following five commonly used success scores: (1) accuracy (Acc), (2) Mathew's Correlation Coefficient (MCC), (3) sensitivity (Sn), (4) specificity (Sp), and (5) area under ROC curve (AUC). As for their rigorous definitions and intuitive formulations, see [21, 23, 24, 35, 38, 60]. Furthermore, the corresponding ROC (receiver operating characteristic) [68] curve is also provided and saved in a PNG file. Finally, by optimizing these scores with respect to all possible parameters, the corresponding best model will be generated.

### Model training

The model is trained with LIBSVM [61] using the RBF kernel. The trained model thus obtained and all its optimized parameters are saved in a separate file, which will be used as the input for “predict.py”.

### Cross validation

Built-in the Pse-Analysis package is also a set of validation operators, which can be used to automatically validate the model from sub-sampling (or K-fold cross-validation) test, and jackknife (or leave-one-out) test, the three most used cross-validation approaches [69].

### Manual of Pse-Analysis

To maximize users’ convenience, the manual of how to use Pse-Analysis is provided, which can be directly downloaded at http://bioinformatics.hitsz.edu.cn/Pse-Analysis/static/download/Pse-Analysis_manual.pdf.

## CONCLUSIONS

Now we are living in a century or era to pursue the goal to minimize various tedious things and leave them to be done by robots or computers, such as in developing autonomous cars or self-driving cars. The present study represents one step forward to such a goal in genome and proteome analyses. It has not escaped our notice that the idea and approach can also be used to many other areas so as to substantially speed up their development accordingly.
